# The complete chloroplast genome sequence of *Manilkara zapota* (Linn.) van Royen

**DOI:** 10.1080/23802359.2019.1623122

**Published:** 2019-07-10

**Authors:** Jin Liu, Sheng-Nan Ren, Kai-Xiong Li, Tong Xu, Ying-Feng Niu, Chao Shi

**Affiliations:** a Yunnan Institute of Tropical Crops, Xishuangbanna, PR China;; b College of Marine Science and Biological Engineering, Qingdao University of Science and Technology, Qingdao, China;; c Kunming Institute of Botany, Chinese Academy of Sciences, Kunming, PR China

**Keywords:** Chloroplast genome, *Manilkara zapota*, Ebenaceae

## Abstract

The complete chloroplast genome sequences of *Manilkara zapota* (Linn.) van Royen in Xishuangbanna, Yunnan province were reported in this study. The length of the sequence was 159,853 bp long with the large single copy (LSC) region of 89,632 bp, the small single copy (SSC) region of 18,747 bp, and two inverted repeat (IR) regions of 27,737 bp. The plastome contained 125 genes, including 84 protein-coding, 8 ribosomal RNA, and 33 transfer RNA genes. The overall GC content was 37.0%. Phylogenetic analysis of 12 representative plastomes within the order Ebenales suggests that *M. zapota* (Linn.) van Royen is closely related to the species in family Ebenaceae.

*Manilkara zapota* (Linn.) van Royen, commonly known as the sapodilla or sapota, is a medium-sized evergreen tree (Lasekan and Yap 2018) which belongs to the family of Sapotaceae. *Manilkara zapota* (Linn.) van Royen is native to Mexico and Central America (Mohanapriya et al. 2018) and now spread throughout regions with tropical climate (Karmee 2017), especially in South East Asia including Southern China. It has high edible and medicinal value (Siddiqui et al. 2018). The fruits are sweet, nutritious and with characteristic delicate flavour, the peel and epicarp were selected for pectin extraction, the seeds were used to prepare oil and the leaf has been traditionally used for the treatment of coughs, cold, dysentery, and diarrhea (Ling et al. 2018). As an important plant, its complete chloroplast genome and phylogenetic evolution based on chloroplast have not yet been reported.

Leaf samples of *M. zapota* (Linn.) van Royen were obtained from Xishuangbanna, Yunnan province in China. After DNA extraction, a library with the insertion size of 400 bp was constructed and high-throughput DNA sequencing (pair-end 150 bp) was performed on an Illumina Hiseq 2500 platform (housed in Kunming Institute of Botany, Kunming, China), generating approximately 3 Gb of sequence data. The specimen of this tree and the isolated DNA were stored in Yunnan Institute of Tropical Crops (YITC), Jinghong, China.

The filtered reads were assembled using the program NOVOPlasty (Dierckxsens et al. 2017). The accurate new annotated complete chloroplast genome was submitted to GenBank with accession number MK790101. The complete chloroplast genome of *M. zapota* (Linn.) van Royen is 159,853 base pairs (bp) in length, containing a large single-copy (LSC) region of 89,632 bp, the small single copy (SSC) region of 18,747 bp, and two inverted repeat (IR) regions of 27,737 bp. The new sequence possesses a total of 125 genes, including 84 protein-coding genes, 8 rRNA genes, and 33 tRNA genes. The overall GC-content of the whole plastome is 37.0%.

To further investigate its phylogenetic position, a neighbor-joining tree was constructed based on complete chloroplast genome sequences of 12 other Sapotaceae species using MEGA7 (Kumar et al. 2016) with 1000 bootstrap replicates. Here, we aligned all 12 sequences using MAFFT (Katoh and Standley 2013). The phylogenetic position of other species is consistent with a previous study (Figure 1).

**Figure 1.**
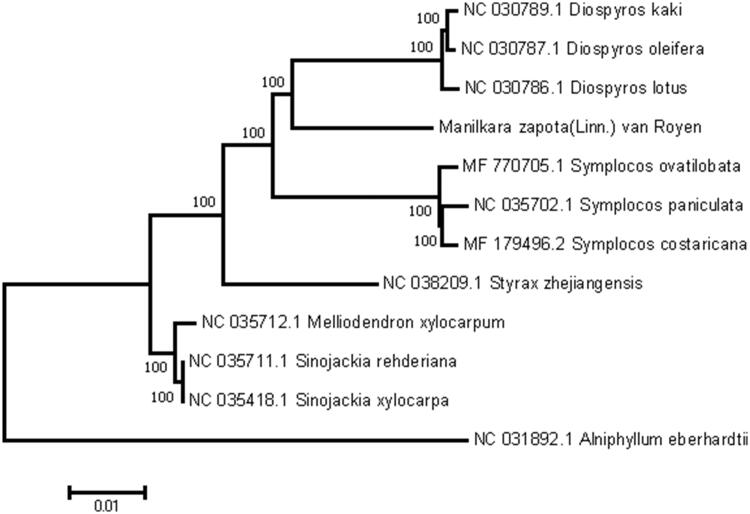
The maximum-likelihood (ML) tree based on the 12 representative chloroplast genomes of order Ebenales. The bootstrap value based on 1000 replicates is shown on each node.
